# Type III restriction endonucleases are heterotrimeric: comprising one helicase–nuclease subunit and a dimeric methyltransferase that binds only one specific DNA

**DOI:** 10.1093/nar/gku122

**Published:** 2014-02-06

**Authors:** Annika Butterer, Christian Pernstich, Rachel M. Smith, Frank Sobott, Mark D. Szczelkun, Júlia Tóth

**Affiliations:** ^1^Biomolecular & Analytical Mass Spectrometry and Center for Proteomics (CFP-CeProMa), Department of Chemistry, University of Antwerp, Antwerp 2020, Belgium and ^2^DNA-Protein Interactions Unit, School of Biochemistry, University of Bristol, Bristol BS8 1TD, UK

## Abstract

Fundamental aspects of the biochemistry of Type III restriction endonucleases remain unresolved despite being characterized by numerous research groups in the past decades. One such feature is the subunit stoichiometry of these hetero-oligomeric enzyme complexes, which has important implications for the reaction mechanism. In this study, we present a series of results obtained by native mass spectrometry and size exclusion chromatography with multi-angle light scattering consistent with a 1:2 ratio of Res to Mod subunits in the EcoP15I, EcoPI and PstII complexes as the main holoenzyme species and a 1:1 stoichiometry of specific DNA (sDNA) binding by EcoP15I and EcoPI. Our data are also consistent with a model where ATP hydrolysis activated by recognition site binding leads to release of the enzyme from the site, dissociation from the substrate via a free DNA end and cleavage of the DNA. These results are discussed critically in the light of the published literature, aiming to resolve controversies and discuss consequences in terms of the reaction mechanism.

## INTRODUCTION

Bacteria and archaea protect themselves from potentially lethal phage infections by expressing various Restriction Modification (RM) systems. These dual-activity enzymes inactivate the invading foreign DNA by generating double strand breaks, while on the other hand modifying (methylating), and therefore protecting the host’s genome from the endonuclease activity (1). Based on their subunit composition, cofactor and recognition site requirements and mechanism of action, these enzymatic systems can be classified as Types I–IV restriction endonucleases (REs) (2,3). The Type III REs are hetero-oligomeric assemblies, comprising polypeptides encoded by the *res* (restriction) and *mod* (modification) genes (4). They require two copies of their unmethylated, short asymmetric recognition site to be present in the target DNA, in an inverted repeat orientation (4,5). DNA cleavage is Mg^2+^- and ATP hydrolysis-dependent and does not require the presence of the cofactor *S*-adenosyl methionine (AdoMet) [although this can play a role as allosteric activator and specificity factor (6)]. The most thoroughly characterized Type III RM enzymes are EcoP15I and the closely related EcoPI both from *Escherichia coli*. Despite several decades of work, there are still controversies regarding the mechanism of DNA cleavage (7,8). One area of dispute is the subunit stoichiometry of the endonuclease complex, which we consider here.

The products of the EcoP15I and EcoPI *mod* genes are 73 and 74 kDa proteins, respectively (Table 1), capable of recognition site binding (5′-CAGCAG-3′ for EcoP15I, 5′-AGACC-3′ for EcoPI) and N6-adenosine methylation using AdoMet as a methyl donor. The Mod subunit when expressed on its own is a stable protein, forming a dimer in solution and works as an independent methyltransferase (9,10). The dimer formation of EcoP15I Mod has been recently supported by data from small-angle X-ray scattering (SAXS) experiments (11). The *K*_D_ for this dimerization must be very low (probably in the sub-nanomolar range) as free Mod monomers are never seen in methyltransferase preparations (12–14).

**Table 1.** Theoretical molecular weights (Da) of Res and Mod subunits and various subunit stoichiometries for EcoP15I, EcoPI and PstII

	Res	Mod	R_1_M_1_	R_1_M_2_	R_2_M_1_	R_2_M_2_
EcoP15I	110 957	74 094	185 051	259 145	296 008	370 102
EcoPI	111 459	73 486	184 945	258 431	296 404	369 890
PstII	108 264	63 715	171 979	235 694	280 243	343 958

The products of the EcoP15I and EcoPI *res* genes are nearly identical ∼111 kDa proteins (Table 1) which are fusions of a Superfamily 2 helicase and a RecB-family endonuclease (15,16). A recent study also identified several regions within the Res subunit (four in the helicase domain and two in the endonuclease domain) capable of non-specific DNA binding (17). In complex with the Mod subunits, Res forms a holoenzyme complex that can hydrolyse ATP and DNA. Attempts to express and purify the unmodified Res subunit without Mod have proven unsuccessful, most likely because the Mod subunit(s) stabilize the Res subunit(s) (18), but a fusion protein (with chitin binding protein) has been purified (12).

In an earlier study (12), the molecular mass of untagged EcoP15I and EcoPI was analysed using analytical ultracentrifugation (AUC) and found to be ∼407 and ∼409 kDa, respectively. These data were interpreted as a Res_2_Mod_2_ heterotetrameric stoichiometry (Supplementary Figure S3). Quantitative densitometric analysis of samples run on SDS–PAGE gels was consistent with this conclusion (12). A more recent work using EcoP15I enzyme supplied by New England Biolabs (NEB) also confirmed a heterotetrameric holoenzyme composition using AUC and dynamic light scattering experiments (11). In the same study the low resolution structure of the EcoP15I endonuclease was determined using SAXS. The data suggested that the holoenzyme has a crescent shape, most likely with the Mod dimer in the centre and one Res subunit at each end [similar to the symmetrical arrangement of HsdR, HsdM and HsdS subunits of the Type I REs (19)]. However, as currently no atomic Type III RE structure is available, only partial homology models could be assembled in the SAXS envelope, indicating a significant level of uncertainty. Although the lowest χ^2^ values were obtained when docking the Res_2_Mod_2_ model in the envelope, other stoichiometries (e.g. Res_1_Mod_2_) cannot be unambiguously excluded based on these data.

In contrast to these two studies above, Wyszomirski *et al.* (17) found two oligomeric species in their His-tagged EcoP15I preparations that could be separated by gel filtration chromatography, with Res:Mod ratios of 1:1 (‘peak 1’) and 1:2 (‘peak 2’), as determined by densitometric analysis of SDS–PAGE gels. As the latter species showed ∼6-fold higher specific activity in DNA cleavage assays (although DNA binding was not included) and 2- to 3-fold higher specific activity in ATPase assays, it was considered as the main active EcoP15I form. The stoichiometry of this fraction was studied further by analysing its aromatic amino acid composition using second derivative ultraviolet (UV) absorption spectroscopy and was found to be Res_1_Mod_2_ (17). This result was also confirmed by gel filtration and analytical ultracentrifugation experiments at low protein concentrations (<0.4 µM), more typical of functional Type III RE assays. Furthermore, the AUC data suggested that the heterotrimer has an elongated shape and/or contains disordered flexible loops. The species in peak 1 proved to be inhomogeneous and prone to aggregation in the AUC experiments; peak 2 also showed signs of oligomerization at high concentrations. It was also reported that when using a low copy number plasmid for expression of a non-tagged enzyme, the preparations yielded the Res_1_Mod_2_ form only. The authors suggest that the species with 1:1 Res:Mod ratio represents a trapped intermediate on the assembly pathway of the active heterotrimer.

Understanding the correct stoichiometry of the Type III RM enzymes is important in unravelling their detailed mechanism of action. First, there is the question of whether one or two Res subunit(s) are required per holoenzyme. In cutting DNA, Type III REs can communicate between sites many hundreds of base pairs apart (5,20). ATP hydrolysis by the Res subunits plays a key role in this communication process. Interpreting how this occurs needs clearer knowledge of whether there are one or two helicases present. Although earlier models for helicase activity suggest a role for dimeric enzymes (21), these have been superseded by schemes in which helicases act exclusively as either monomers or hexamers/heptamers (22). Second, subsequent DNA cleavage requires the interaction of two holoenzymes (12). Thus, there may be either two or four endonuclease domains present during the production of a single double-stranded DNA break.

Third, the subunit stoichiometry is also an important question considering the reaction mechanism of methylation. Methyltransferases are active as both monomers and dimers (23,24). Having two Mod subunits per complex raises the possibility of binding two target sequences at the same time, giving rise to DNA looping by either the isolated methyltransferase or the holoenzyme. Although DNA looping is not a necessity for DNA cleavage (25), it may play a role in the assembly of a more stable complex (26,27). Type III recognition sequences are only methylated on one strand (hemimethylation) so dimerization does not play a clear catalytic role in DNA methylation. The interaction of two holoenzymes during cleavage also brings together four Mod subunits, an apparent surfeit given the number of DNA sites (one or two depending on the interaction model). However, a dimeric methyltransferase may only bind one DNA site if either dimerization or DNA binding induces asymmetry in the complex. Understanding how the Type III enzymes bind DNA would also provide a useful model for comparison with other dimeric methyltransferases.

Due to the conflicting data in the literature on the subunit composition of Type III REs, we carried out experiments using native mass spectrometry (native MS) and size-exclusion chromatography (SEC) with multi-angle light scattering (SEC-MALS), techniques that preserve the native, untagged complexes and are not sensitive to the shape of the molecule. Our data are consistent with the heterotrimeric Res_1_Mod_2_ stoichiometry. We also show that despite two target recognition domains being present per enzyme molecule, Type III REs bind only one recognition site at a time. We discuss our results in light of suggested models for the activity of Type III RM enzymes.

## MATERIALS AND METHODS

### Reagents

Chemicals and reagents were purchased from Sigma-Aldrich unless stated otherwise. The term ‘apo’ refers to the nucleotide binding pocket being unoccupied, not to the DNA-binding site in the target recognition domain.

### Materials, protein preparations and DNA oligonucleotides

Type III REs were prepared as reported previously (28). In brief, wild-type (wt) EcoP15I and wt EcoPI enzymes were produced in NovaBlue cells (Novagen) transformed with pUC18-based expression vectors coding the entire Type III RE operon driven by its natural promoter. Following sonication and ultracentrifugation, the crude cell extract was passed through a DEAE-Sepharose (ion exchange), a heparin (affinity) and finally a Superdex 200 (gel filtration) column, selecting for fractions enriched in the given Type III RE. Preparations typically yielded 10–15 mg/l of culture. The commercially available EcoP15I was from NEB (catalogue no: R0646S) supplied as a 10 000 U/ml solution in 10 mM Tris–HCl pH 7.4, 100 mM NaCl, 1 mM DTT, 0.1 mM EDTA, 200 μg/ml Bovine Serum Albumin (BSA) and 50% (v/v) glycerol. The biotinylated EcoP15I was generated by engineering an Avi-tag to the C-terminus of the Res subunit and co-expressing the protein with biotin ligase. The resulting labelled protein was affinity purified on SoftLink Soft Release Avidin resin (Promega) followed by ion exchange using a Mono-Q column (28). The R534A mutant of EcoP15I was generated by Quickchange mutagenesis (Stratagene) using the following oligonucleotides: R534A top strand: 5′-CTTCAAGAGGTAGGGGCCGGTTTGCGTCTTCC-3′, R534A bottom strand: 5′-GGAAGACGCAAACCGGCCCCTACCTCTTGAAG-3′ and was purified identically to the wild-type. We note that all our EcoP15I (wt and mutant) and EcoPI preparations eluted reproducibly as a single peak from all three columns used. PstII was a kind gift from Alice Sears and was expressed in TOP10 cells and purified on a heparin column followed by a Mono-Q column (29). SDS–PAGE gels also confirmed that there was no significant amount of degradation products present in our enzyme preparations (Supplementary Methods and Supplementary Figure S1). Enzyme concentrations were calculated using the theoretical extinction coefficients according to the Res_1_Mod_2_ stoichiometry.

Oligonucleotides were purchased from Eurofins MWG, with the top strand oligonucleotides either unlabelled or 5′-labelled with hexachlorofluorescein (Hex) via a 12C linker. Two specific DNA (sDNA) duplexes carrying the Type III RE recognition sequences (5′-CAGCAG-3′ for EcoP15I, 5′-AGACC-3′ for EcoPI) and a non-specific DNA (nsDNA) duplex were used in this study (Figure 2A and Supplementary Figure S4A). The duplexes were 50 bp in length, carrying the recognition sites six bases from the 5′-end where applicable. For annealing the duplexes, oligonucleotides were mixed at 10 µM concentration in TE (10 mM Tris–HCl pH 8.0, 1 mM EDTA) at a 1:1 ratio in the case of unlabelled DNA or at a 1.3:1 in the case of labelled DNA. Oligonucleotide mixtures were heated to 96°C for 4 min and allowed to cool to room temperature overnight.

The assay buffer used throughout the experiments (Standard Assay Buffer) was the following: 50 mM Tris–HCl pH 8.0, 50 mM KCl, 10 mM MgCl_2_, 1 mM DTT and 0.1 mg/ml BSA. All reactions were carried out at room temperature (20 ± 1°C).

### Size-exclusion chromatography with multi-angle light scattering

Protein samples (0.5–1.0 mg/ml, 1.25–2.5 µM) were run in Standard Assay Buffer minus BSA at 1 ml/min on a Superdex 200 analytical size-exclusion column (GE Healthcare), attached to a light scattering diode array and a differential refractive index detector (Wyatt Technology UK Ltd.). This setup allows for the accurate measurement of absolute molecular masses of elutes or even a mixture of complexes, independent of shape, calculated from the ratio of light scattering and the differential refractive index. Chromatograms were analysed using the ASTRA software (v6.0.5.3, Wyatt Technology UK Ltd.). Where indicated, protein samples were incubated with various molar ratios of Hex-labelled specific 50-mer DNA duplexes for at least 30 min before the chromatography run. BSA was omitted from the standard assay buffer to avoid possible difficulties with peak identification. For samples with 1 mM ATP (Sigma), the running buffer was also supplemented with the nucleotide.

**Figure 1.**
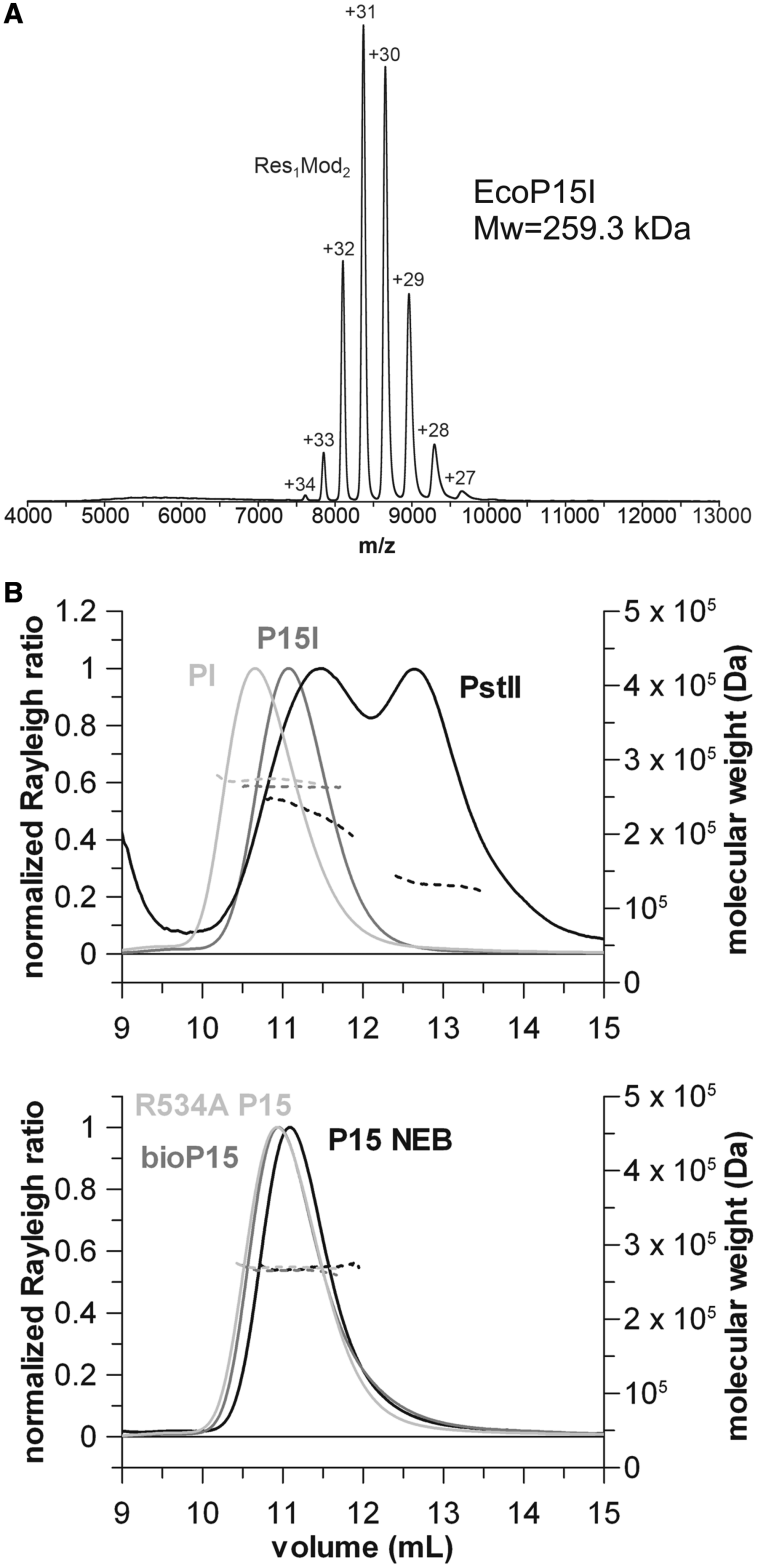
Evidence for the heterotrimeric stoichiometry of Type III REs. (**A**) Native mass spectrum of wt EcoP15I. The Y-axis is relative intensity, scaled to the most intense peak in the spectrum, which is the 31+ charge state of the enzyme complex. The numbers above the peaks are the measured charge states for the given molecular species. The observed molecular weight (259.3 kDa) corresponds closely to that expected of the Res_1_Mod_2_ heterotrimer (259145 Da, Table 1). (**B**) SEC-MALS traces of various Type III REs. All enzymes yielded a single elution peak with a molecular weight supporting the Res_1_Mod_2_ subunit stoichiometry, except for PstII, which eluted as a double peak indicating a mixture of Mod_2_ and Res_1_Mod_2_ species. Solid lines represent the normalized light scattering (left Y-axis), whereas dotted lines show the calculated molecular masses (right Y-axis). Traces are shown in two separate graphs for clarity, noting that the wt EcoP15I and EcoP15I obtained from NEB had the same elution volume. See “Evidence for a Res1Mod2 stoichiometry for Type III REs” in the Results section and Table 3 for further details.

**Figure 2.**
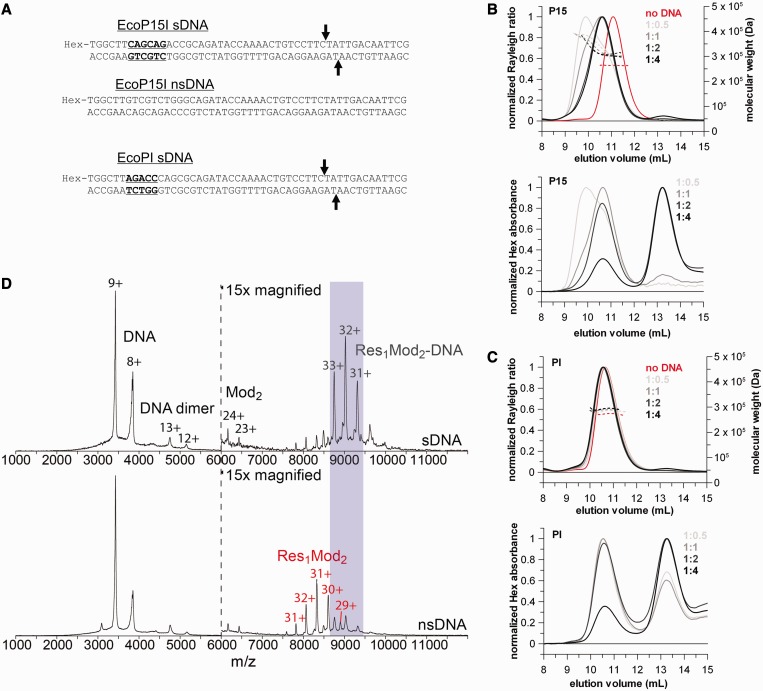
The heterotrimer Type III REs bind a single DNA with a specific site despite the presence of two target recognition domains. (**A**) Sequence of the specific (sDNA) and non-specific ns(DNA) 50-mer DNA duplexes. The 5′-CAGCAG-3′ and 5′-AGACC-3′ recognition sites for EcoP15I and EcoPI, respectively, are highlighted with bold and underlined fonts. The cleavage positions (25 and 27 bases downstream of the recognition site in the top and bottom strand, respectively) are marked with arrows. (**B** and **C**) SEC-MALS traces of a constant amount of EcoP15I (B) or EcoPI (C) with sub-stoichiometric amounts to saturating excess of the relevant specific 50-mer DNA duplex (ratios are enzyme:DNA). In the upper graphs, solid lines represent the normalized light scattering (left Y-axis), whereas dotted lines show the calculated molecular masses (right Y-axis). In the lower graphs, lines show the hexachlorofluorescein (Hex) absorbance traces of the same SEC-MALS runs to confirm the presence of DNA in the eluted complexes. (**D**) Native mass spectra of wt EcoP15I pre-incubated with a 2-fold excess of specific (top panel) and non-specific (bottom panel) 50-mer duplex DNA. The Y-axis is relative intensity, scaled to the most intense peak in the spectrum, which is the 9+ charge state of the free DNA. Peaks on the left hand side of the dotted vertical line correspond to free excess DNA, whereas peaks on the right hand side can be identified as Res_1_Mod_2_ heterotrimers in complex with a single copy of specific DNA or free Res_1_Mod_2_ holoenzyme in the case of non-specific DNA. As the protein ionizes much less efficiently than the DNA, the protein and protein–DNA peaks have been magnified ×15 relative to the DNA-alone peaks for presentation purposes.

**Figure 3.**
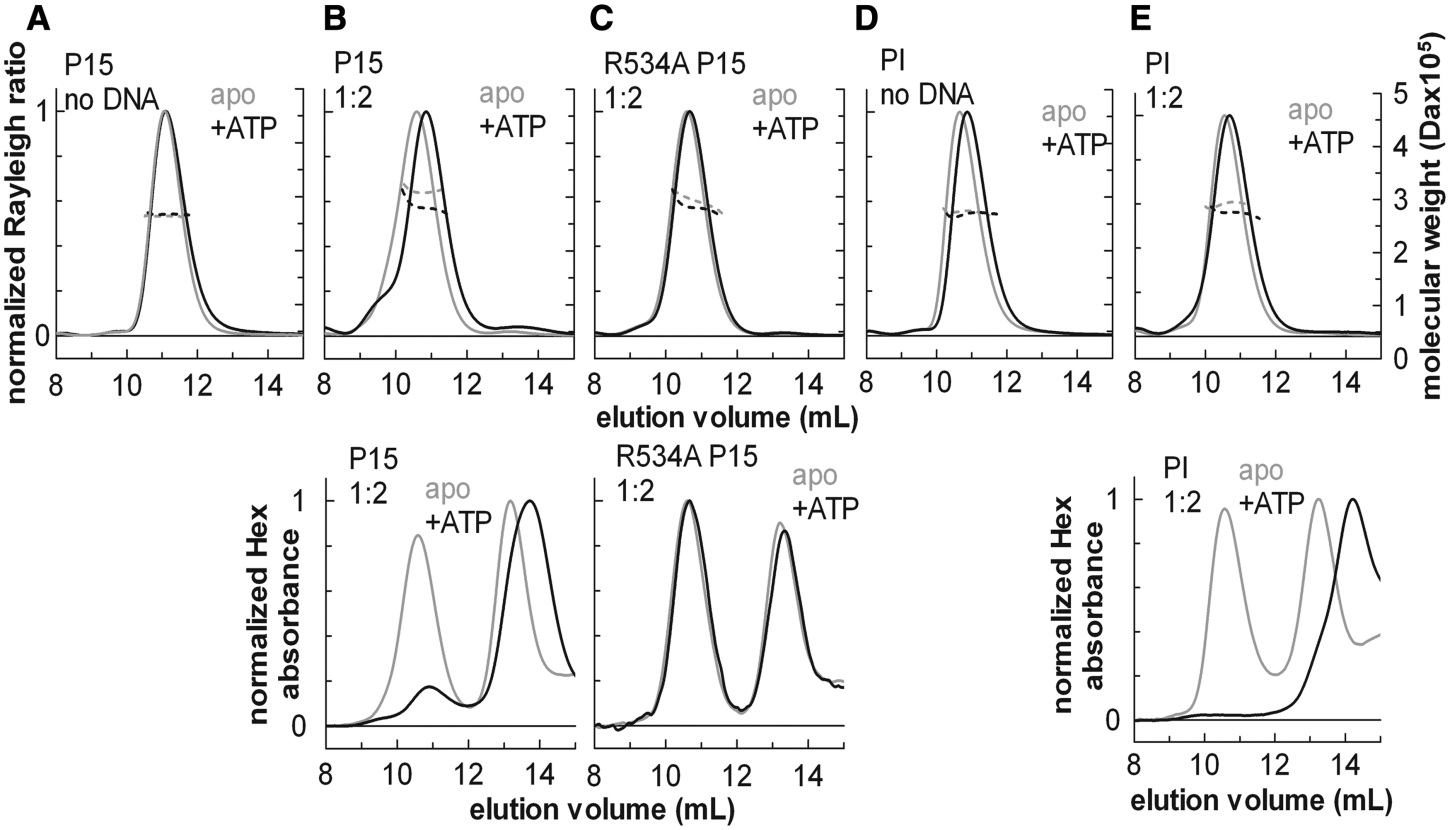
SEC-MALS analysis of the effect of ATP on the protein stoichiometry and DNA binding. SEC-MALS traces of wt EcoP15I (P15), EcoP15I helicase mutant (R534A P15I) or wt EcoPI (PI), with (black lines) or without ATP (grey lines), in the absence (apo) or presence of a 2-fold excess of specific 50-mer DNA duplex (1:2). In the upper graphs, solid lines represent the normalized light scattering (left Y-axes), whereas dotted lines show the calculated molecular masses (right Y-axes). In the lower graphs, lines show the hexachlorofluorescein (Hex) absorbance traces of the same SEC-MALS runs to confirm the presence of DNA in the eluted complexes, where included. The SEC-MALS profiles are not affected significantly by ATP in the absence of specific DNA (**A**, **D**). A significant shift in the elution volume and/or a reduction of the calculated mass is indicative of a conformational change and dissociation from the specific DNA (**B**, **E**). The elution volume of the R534A mutant does not change in the presence of ATP and specific DNA (**C**). Note that for the wt enzymes, the elution volume of the free DNA is significantly increased in the presence of ATP (**B**, **E**), suggesting that the DNA is cleaved by the enzymes (see “ATP hydrolysis releases the Type III holoenzyme from DNA without affecting its stoichiometry” in the Results section and Supplementary Figure S4).

To check for reproducibility of the chromatography during the SEC-MALS runs, we measured BSA as a standard at the beginning and end of each measurement day and the elution volumes within the same reaction conditions (e.g. in the absence of ATP) were extremely reproducible (with an average and standard deviation (SD) elution volume of 13.74 ± 0.01 ml). The inclusion of ATP in the samples and the running buffer caused a slight but reproducible shift (+0.1 ml) in the elution volume of BSA. Figures 1B, 2B and C and 3 show the traces of single chromatography runs rather than averages.

### Native MS

For enzyme-only measurements, samples (10 µM) were dialysed against 100 mM ammonium acetate pH 7.0 prior to analysis. In the case of enzyme–DNA complexes, they were mixed in the enzyme storage buffer [10 mM Tris–HCl pH 8.0, 100 mM NaCl, 1 mM DTT and 50 % (v/v) glycerol] at a 1:2 ratio (5 µM enzyme and 10 µM DNA) and magnesium acetate added to the samples at 2 mM. For experiments with ATP, 4 mM MgOAc and 2 mM ATP were added. Following 5 min incubation, the samples were buffer-exchanged into 100 mM ammonium acetate using Biorad Micro Bio-Spin P-6 Gel Columns to get rid of excess magnesium and other non-volatile buffer components that would interfere with the measurement. Enzyme stoichiometry measurements were performed on a Q-TOF II instrument (Waters) modified for high-mass operation (MSVision; including vacuum adaptations, 32 k quadrupole and extended voltage range) (30,31). Commercially available capillaries (PicoTip, New Objective) were used for nano-electrospray ionization with a spray voltage of 1.6 kV and source temperature of 80°C. Gas pressures were 4, 2.3 and 1.2 × 10^−2 ^mbar for backing, source and collision cell, respectively. Tuning parameters were chosen with the aim to preserve non-covalent interactions (voltages were 80, 10 and 60 V for sample and extractor cone and collision energy, respectively). For enzyme–DNA complexes, a Waters Synapt G2 HDMS was used with the following parameters: pressures, backing 5 mbar, source 5.8 × 10^−3 ^mbar, trap 4.4 × 10^−2 ^mbar; voltages, nano-electrospray using in-house-made gold-coated borosilicate capillaries 1.7–1.9 kV, sampling and extractor cone 60 V and 1 V, trap and transfer collision energy 50–60 V and 2.1 V, trap DC bias 50 V and a source temperature of 30°C.

## RESULTS

To provide further evidence for the quaternary organization of the Type III holoenzymes, we chose to use two techniques not previously applied to these enzymes and which can give accurate determination of protein size largely independent of overall molecule shape: (a) Native MS, which can analyse several species in a single spectrum and can provide quite rich detail about complex molecular mixtures without the necessity for extensive model fitting or assumptions (e.g. as is necessary for AUC) (32–34). One shortcoming of this approach is that, for technical reasons, the buffer composition is different to the standard Type III reaction buffer (‘Materials and Methods’ section). However, it is standard practice in native MS to replace other buffers with a compatible, volatile buffer such as ammonium acetate, which is chemically very similar to potassium chloride. The concentration and pH of this buffer can be chosen to match the requirements of the protein and other key components (such as, e.g. DTT or Mg^2+^) can be retained. A large body of evidence in the literature demonstrates that this MS buffer does not typically affect the stoichiometry of protein complexes; (b) SEC-MALS equipped with a Wyatt light scattering diode array and a differential refractive index detector, which measures molar mass directly following separation of different species by conventional SEC using Superdex 200 media (35). Although less accurate than native MS, SEC-MALS allowed us to use our standard reaction buffer, which supports the full enzyme reaction. Note that the technique is limited by sample heterogeneity following SEC, and the fairly large dilutions during SEC which could potentially alter the equilibrium mixtures present in the samples. We tested untagged EcoP15I and EcoPI samples from a number of sources, including a commercial stock from New England Biolabs (‘Materials and Methods’ section) as well as a biotin-tagged EcoP15I used in our previous single-molecule studies (28). We also tested a sample of PstII holoenzyme, a more distantly related Type III RM enzyme. Molecular weights for different theoretical quaternary structures are given in Table 1 for reference. To test DNA binding, we utilized 50 bp specific DNA duplexes used in previous studies that support specific binding and ATP hydrolysis (28) and an equivalent non-specific duplex (Figure 2A and Supplementary Figure S4A). Theoretical molecular weights for the binding of one or two DNAs by different holoenzyme species are given in Table 2 for reference.

**Table 2.** Theoretical molecular weights (Da) of various subunit stoichiometries of EcoP15I, the biotinylated EcoP15I and EcoPI in 1:1 and 1:2 complex with Hex-labelled or unlabelled 50-mer specific DNA

		Hex-labelled 50-mer		unlabelled 50-mer	
		1:1	1:2	1:1	1:2
**EcoP15I**	***R_1_M_2_***	290 658	322 171	289 914	320 683
	***R_2_M_2_***	401 615	433 128	400 871	431 640
	***M_2_***	179 701	211 214	178 957	209 726
					
**bio-EcoP15I**	***R_1_M_2_***	292 989	324 502	292 245	323 014
	***R_2_M_2_***	406 277	437 790	405 533	436 302
	***M_2_***	179 701	211 214	178 957	209 726
					
**EcoPI**	***R_1_M_2_***	289 944	321 457	289 200	319 969
	***R_2_M_2_***	401 403	432 916	400 659	431 428
	***M_2_***	178 485	209 998	177 741	208 510

### Evidence for a Res_1_Mod_2_ stoichiometry for Type III REs

To check for the quality of our Type III RE preparations, we ran samples on an SDS–PAGE gel, which confirmed that the enzymes are highly homogeneous and there is no significant proteolytic degradation in the purified samples (Supplementary Methods and Supplementary Figure S1). Native MS of EcoP15I, EcoPI and biotinylated EcoP15I showed in all cases almost exclusively one species with a Res_1_Mod_2_ stoichiometry (Figure 1A and Supplementary Figure S2), in agreement with the enzymes eluting as a single peak during purification. For all three enzyme complexes a charge state distribution from +27 to +34 was measured with the most intense peak being always the +31 charge state. These charge state distributions indicated a similar shape for the different enzymes, as the amount of charge a protein accumulates during the electrospray process is roughly proportional to the solvent accessible surface area for most globular, folded proteins (32,36). Experimentally determined masses were 259.3 kDa for EcoP15I and 258.5 kDa for EcoPI, respectively. The biotinylated EcoP15I gave a mass of 261.5 kDa. All these values are in close accordance with the theoretical masses for the Res_1_Mod_2_ heterotrimeric complexes.

Following SEC of the EcoP15I and EcoPI enzymes, we reproducibly obtained peaks consistent with single monodisperse species in MALS experiments (Figure 1B). The determined molecular weights could be most confidently interpreted as a Res_1_Mod_2_ heterotrimer (Table 3). The calculated values did not change significantly, and thus did not affect the interpretation of the molecular masses, when a wider peak selection was used during data analysis. Moreover, if Res_2_Mod_2_ becomes Res_1_Mod_2_ due to sample dilution during the SEC run, there must be release of free Res which we would get as a separate peak—however we did not observe additional peaks. Importantly, our EcoP15I preparations of various sources showed the same molecular mass as the commercially available EcoP15I from New England Biolabs suggesting that the fine details of the preparation protocol most likely do not affect the stoichiometry of the untagged enzyme. We repeated the chromatography runs under different buffer conditions (modified NEBuffer4: 20 mM Tris–acetate, pH 7.9, 10 mM CaCl_2_, 50 mM potassium acetate and 1 mM DTT) and got the same results (data not shown). For the apo EcoP15I in the absence of ATP the mean and SD elution volumes for four runs were 11.01 ± 0.08 ml, using different enzyme preparations including a commercial one from NEB, indicating that the chromatography runs were highly reproducible. All the data shown are from runs in the Standard Assay Buffer to keep reaction conditions consistent across different assays. Changes to EcoP15I, such as the addition of a biotin-avidin tag at the C-terminus of the Res subunit [used earlier in our single-molecule sliding assay (28)], or introducing the R534A point mutation (rendering the enzyme ATP hydrolysis deficient) does not influence the stoichiometry. We also observed that EcoPI reproducibly eluted earlier from the size exclusion column than EcoP15I.

**Table 3.** Molecular weights (kDa) of free and 50-mer specific DNA complexed Type III REs in the absence and presence of ATP as measured by SEC-MALS experiments

	− ATP					+ ATP				
	no DNA	1:0.5	1:1	1:2	1:4	no DNA	1:0.5	1:1	1:2	1:4
EcoP15I	264	304	306	311	306	267	279	274	280	
		373^b^	377^b^						
EcoP15I NEB	269									
bioEcoP15I	266									
R534A EcoP15I	270			294		269			300	
EcoPI	273	283	289	291	293	268	277		275	
PstII	132^a^									
	205^a^

^a^Equilibrium between two species.

^b^Equilibrium between multiple species, average molecular weight of multiple species due to sample heterogeneity.

The SEC of the PstII sample produced two broad peaks as observed previously in the absence of Triton X-100 (37). According to the molar masses derived from the MALS, the sample is an equilibrium mixture of Res_1_Mod_2_ and Mod_2_ species, although higher order species cannot be ruled out explicitly. The analysis of the previous SEC data (37) relied on calibration using size standards and on the relative amounts of Res and Mod determined using quantitative densitometry following SDS–PAGE. This gave ambiguous results, with a retention volume midway between theoretical Res_1_Mod_2_ and Res_2_Mod_2_ species but with a significant excess of Mod over Res. We favoured a Res_2_Mod_2_ species based on our previous analysis of EcoPI and EcoP15I (12), although we did not rule out the possibility that the Res_1_Mod_2_ species existed. Our new data does not alter the overall conclusions of Sears and Szczelkun (37), that subunit assembly modulates the activities of PstII, something that was also concluded by Wyszomirski *et al.* (17) using EcoP15I.

Previous sedimentation equilibrium (SE) and sedimentation velocity (SV) AUC experiments that were interpreted as showing a 1:1 Res:Mod ratio used proteins prepared by alternative protocols to those used here (11,12). To check how the protein preparations used in this study behaved during ultracentrifugation, we revisited the SE AUC experiments (Supplementary Methods and Supplementary Figure S3). Depending on the protein concentration used and the rotor speed applied, variable molecular weights were obtained that are in between that of the theoretical Res_1_Mod_2_ and Res_2_Mod_2_ species or, in some cases, even above the expected molecular weight for the Res_2_Mod_2_ species (Table 4). This indicates that, probably due to the characteristics of the technique, SE AUC gives ambiguous results, possibly reporting on association/aggregation processes over the long running times of the experiment (see ‘Discussion’ section). SV AUC experiments were performed by Janscak *et al.* (12) previously and gave the same 1:1 ratio as their SE experiments, suggesting that this approach is subject to similar limitations.

**Table 4.** Calculated molecular weights (kDa) of wt EcoP15I obtained by analytical ultracentrifugation at multiple protein concentrations at various running speeds. The molecular mass values were obtained by fitting a single ideal species model to the experimental data (see also Supplementary Methods and Supplementary Figure S3)

EcoP15I concentration	6 k	7.5 k	10 k	Average of three speeds
0.093 mg/ml	349.0	324.7	305.0	318.5
0.23 mg/ml	344.6	325.9	298.5	314.7
0.46 mg/ml	393.0	392.8	345.6	366.8
Average of three concentrations	376.0	363.5	330.1	347.6

### EcoPI and EcoP15I holoenzyme can only bind one DNA molecule at a time

As all the tested Type III REs contain two Mod subunits, the heterotrimeric enzymes could, in theory, simultaneously bind two molecules of DNA containing the recognition sequence. To look into this, we carried out a systematic DNA titration from sub-stoichiometric DNA concentrations to saturating DNA concentrations using EcoP15I and EcoPI and examined the samples first using SEC-MALS (Figure 2B and C; Table 3). A Hex-labelled DNA was used (Figure 2A) to independently monitor the DNA species within the retention volumes. ATP was excluded so that only DNA binding would be measured (and not motion on DNA or DNA cleavage).

Following SEC, EcoP15I and EcoPI showed different profiles as a function of DNA concentration. At sub-stoichiometric concentrations, EcoP15I seems to be in equilibrium between a Res_1_Mod_2_ complex bound to one DNA molecule (304 kDa) and a less well-defined species with a putative molecular mass of ∼373 kDa (Figure 2B and Table 3). This latter species is still visible at equimolar DNA, but disappeared as we increased the DNA concentration of the specific DNA further. It might represent a state where EcoP15I undergoes a conformational change upon binding to the recognition site and exposes a protein–protein interaction surface that can recruit other proteins from free solution. However, the estimated molecular weight of this species is less reliable due to the sample heterogeneity following SEC and does not correspond clearly to a straightforward combination of enzyme(s) and DNA based on values in Tables 1 and 2. We suggest that an interaction occurs between DNA-bound species and free protein(s), although the complexes are clearly heterogeneous based on the light scattering traces. Using excess DNA over holoenzyme, only a single species was observed with a more defined molecular weight of 306–311 kDa (Figure 2B and Table 3), consistent with one molecule of DNA bound to a Res_1_Mod_2_ complex (Table 2). This suggests that, in the absence of free protein, the above mentioned interaction does not appear to occur between DNA-bound species alone. Under the conditions used, we could not drive the formation of a complex with two molecules of DNA bound to the heterotrimer.

In contrast, the different EcoPI complexes formed during the DNA titration eluted with the same volume (within experimental error) following SEC (Figure 2C). A species with a putative average molecular mass of ∼373 kDa equivalent to that seen with EcoP15I was not observed with sub-stoichiometric DNA, suggesting that the formation of this complex by EcoP15I is most probably not an important mechanistic feature of all Type III REs. The molar masses derived from the EcoPI MALS data are also consistent with only one molecule of DNA bound to a heterotrimer (Tables 2 and 3) and the Hex traces also confirm the presence of DNA in the eluates. We also note that there is a significant amount of free DNA present at the sub-stoichiometric DNA sample compared with the EcoP15I binding experiments.

In native MS experiments, an enzyme:DNA ratio of 1:2 was used (Figure 2D). Similar results were obtained when the ratio was increased to 1:12 (data not shown). The experimentally determined masses were 290.4 kDa for the EcoP15I–DNA complex and 288.1 kDa for the EcoPI–DNA complex (data not shown). For the biotinylated EcoP15I–DNA complex, a mass of 291.0 kDa was measured (data not shown). These data are all in close agreement with the theoretical masses for a heterotrimer binding to only a single DNA molecule (Table 2). We note that free DNA was visible in the spectra in a larger amount than one would have expected at 2-fold excess of DNA over enzyme. This may be because the DNA ionizes more efficiently than the protein complex and/or the binding is less efficient in the native MS buffer than in our standard reaction buffer. We also see some free Res_1_Mod_2_ and Mod_2_ complexes in relatively low amounts that are most probably due to degradation of the sample during MS. Given that release of a Mod_2_ complex was not observed in the SEC-MALS, we do not think that it reflects a normal reaction intermediate. As shown in the bottom panel of Figure 2D, the amount of free enzyme increased dramatically when non-specific DNA was used, as only a small amount of enzyme seems to bind to this DNA under our experimental conditions.

### ATP hydrolysis releases the Type III holoenzyme from DNA without affecting its stoichiometry

Based on single-molecule and ensemble biochemical assays, we have proposed a model for long-range interaction by Type III restriction enzymes in which ATP hydrolysis acts as a molecular switch that causes dissociation from the site into a fast DNA sliding mode (28). On short DNA oligonucleotides, such as those used above (Figure 2A), this rapidly leads to dissociation from the DNA by sliding off via the DNA ends. One question we sought to address here was, whether the addition of ATP would cause the anticipated DNA release and whether this process was accompanied by changes in the stoichiometry of the holoenzyme. In the absence of DNA, wt EcoPI, wt EcoP15I and R534A EcoP15I (mutated in Motif VI of the helicase, therefore, lacking ATPase activity) had the same heterotrimeric assembly and elution volumes following SEC both with or without ATP (Figure 3A and D), suggesting that in the absence of DNA either these enzymes cannot bind ATP, or that binding ATP alone does not affect the subunit stoichiometry or cause a large conformational change. We note that we do not have any direct evidence for ATP binding by the wt enzymes in the absence of DNA. Type III REs might not bind ATP efficiently in the absence of DNA, as they are very weak ATPases when not bound to DNA (5).

However, in the presence of specific DNA, the elution profiles for the wt EcoP15I altered upon the addition of ATP (Figure 3B), consistent with changes in DNA-binding affinity. For EcoP15I, a broad peak with a shoulder was observed with an elution volume intermediate of that of the free complex with ATP and the DNA-bound complex in the absence of ATP (the shifts being greater than the experimental uncertainty in elution volume of ± 0.1 ml—see ‘Materials and Methods’ section). The population was more heterogeneous and thus the molecular mass less accurate, but the calculated range (274–280 kDa) is in between that of the free enzyme (264 kDa) and the 1:1 enzyme:DNA complex (306 kDa) (Table 3). The Hex absorbance profiles show that addition of ATP causes release of DNA, although some DNA remains associated with the protein peak. This likely represents enzymes that have rebound to the site and have yet to undergo the conformational switch. For the R534A EcoP15I mutant which cannot hydrolyse ATP, no significant change in the elution profiles was observed upon addition of ATP (Figure 3C). We can therefore interpret the changes seen with wt EcoP15I as dissociation of the enzymes from DNA following ATP hydrolysis (see below for comments on the change in DNA elution volume). We did not observe any peaks corresponding to higher or lower order species.

For EcoPI, the addition of ATP did not produce a noticeable shift in the elution profiles (Figure 3D and E). Given that the free and DNA-bound species have the same elution volume, this is not surprising. However, there was a clear loss of the Hex absorbance from the protein peak, indicating the release of the DNA. The molecular mass calculated from the MALS before and after ATP (Table 3) is consistent with release of the DNA without any significant change in protein stoichiometry. We did not observe any peaks corresponding to higher or lower order species. Based on these data, we suggest that the Type III enzyme conformation switch following ATP hydrolysis is not accompanied by changes in holoenzyme stoichiometry. Therefore, the species that dissociates from the site and starts to slide is the Res_1_Mod_2_ species, as expected, based on our single-molecule data where the sliding enzyme can rebind the site (presumably because the Mod subunits remain associated) (28).

We observed for both enzymes that the elution volume of the free DNA is significantly different to that in the absence of ATP (Figure 3B and E, lower panels). One possibility is that this is due to the DNA oligoduplex getting cleaved, as under the above reaction conditions (high enzyme and DNA concentrations and extended incubation times) secondary cleavage can occur (6). This was further corroborated by the fact that the R534A mutant did not produce such a shift (ATP hydrolysis being required for cleavage). To examine this further we collected samples following SEC and separated the DNA species by native TBE–PAGE (Supplementary methods), using both ethidium bromide and Hex fluorescence to observe the DNA (Supplementary Figure S4B). The results confirm that under these reaction conditions, the short single-site DNA is being cleaved by the wt enzymes. The kinetics of the cleavage reaction is slower than dissociation from the site into the sliding state (Supplementary Figure S4C) (also confirmed by Júlia Tóth, unpublished results). We therefore suggest that cleavage arises due to an enzyme bound to the DNA associating *in trans* with a recently activated enzyme that is still in the sliding conformation (either DNA bound or free).

**Figure 4.**
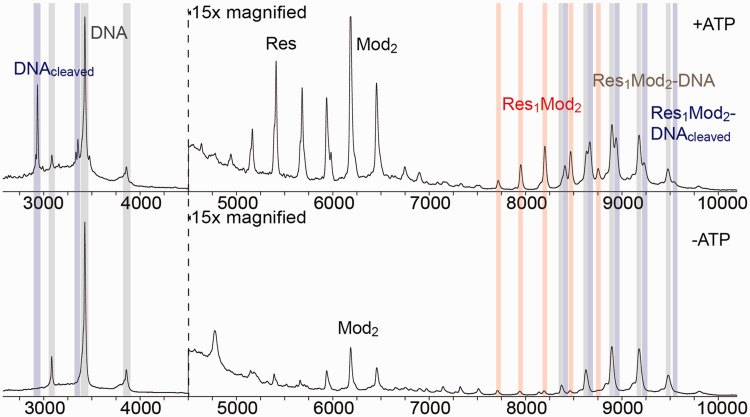
Native MS analysis of the effect of ATP on the protein stoichiometry and DNA binding. Native mass spectra of wt EcoP15I pre-incubated with specific 50-mer DNA duplex (Figure 2) in the presence of ATP (top panel) or absence of ATP (lower panel). The Y-axis is relative intensity, scaled to the most intense peak in the spectrum (the 9+ charge state of the free DNA). Due to lower ionization efficiency, peaks containing protein (all peaks rightward of the dotted vertical line) were magnified ×15 for presentation purposes. Fragments containing the intact DNA, cleaved DNA and the Res_1_Mod_2_ heterotrimer are labelled with grey, blue and red vertical columns, respectively. In the absence of ATP, most of the enzyme forms a stable complex with the specific DNA that partially dissociates to the heterotrimer enzyme and free DNA upon ATP hydrolysis. DNA cleavage occurs in the presence of ATP only. The amount of free Res and free Mod_2_ increases significantly in the presence of ATP probably due to the conformational change associated with sliding (28) that renders the heterotrimer less stable under the native MS conditions.

To confirm the SEC observations, we carried out native MS experiments with EcoP15I pre-incubated with 2-fold excess of sDNA in the presence (Figure 4 upper panel) or absence of ATP (Figure 4 lower panel). In the absence of ATP, the majority of the heterotrimeric enzyme formed a 1:1 complex with a single specific DNA molecule. A small amount of free Mod_2_ was also observed in the samples as well as fragments corresponding to free excess DNA. Upon the addition of ATP, characteristic changes were noted in the mass spectrum. First, fragments originating from free cleaved DNA and the Res_1_Mod_2_ heterotrimer bound to a single cleaved DNA fragment appeared. Moreover, we also observed a marked increase in the peaks corresponding to free Res_1_Mod_2_ heterotrimer indicative of the partial dissociation of the holoenzyme–sDNA complex. We also note that the traces revealed an increased level of free Res and free Mod_2_ subunits in the ATP sample. To check for potential changes in the subunit stoichiometry (i.e. the dissociation of the heterotrimer to Res and Mod_2_) following the conformational switch to the sliding state we also subjected enzyme samples with sDNA and ATP to gel filtration and heparin chromatography and ran the resulting fractions on SDS–PAGE gels (data not shown). We did not detect any change in the Res_1_Mod_2_ heterotrimeric assembly upon ATP hydrolysis. In combination with the SEC-MALS data above, we suggest that the dissociation of the heterotrimer is a consequence of the enzyme being less stable in the sliding conformation under the native MS conditions.

## DISCUSSION

As contradicting observations have been published in the literature regarding the subunit stoichiometry of Type III REs, we carried out native MS and SEC-MALS experiments to determine the molecular masses of these enzyme complexes with improved accuracy. These data strongly support a Res_1_Mod_2_ heterotrimer stoichiometry as recently proposed by Wyszomirski *et al.* (17). We did not notice any difference between untagged and biotin-tagged protein preparations produced by different protocols, labs or strains. We note, however, that tagging the protein with an N-terminal His tag and/or changing the expression yields by using different promoters can produce additional species with subunit stoichiometries other than Res_1_Mod_2_, as seen before by Wyszomirski *et al.* (17). In addition, we were able to determine that the holoenzyme only binds a single specific DNA which is released following ATP hydrolysis without any change in subunit stoichiometry (at least to the majority of enzymes under standard reaction conditions). These combined results simplify the framework for the future consideration of mechanistic models for Type III REs (see below).

The disagreements with previous studies may reflect limitations of the experimental techniques used. We repeated a sedimentation equilibrium AUC analysis of EcoP15I (Supplementary data) and observed significant variations in the estimated molecular mass with protein concentration and centrifugation conditions (Table 4). Similar variations were noted by Reuter *et al.* and the upper end of the estimated values we observed match those reported in Janscak *et al.* (12). We also analysed our Coomassie-stained protein gels using densitometric analysis. Assuming a uniform dye binding, we applied a molecular weight-dependent correction factor and obtained results that were consistent with a Res_1_Mod_2_ stoichiometry (data not shown). The simplest explanation of the different result seen in Janscak *et al.* is that the earlier protein preparations might have contained unbound Res following purification (akin to ‘peak 1’ from Wyszomirski *et al.*). We would argue that the native MS and SEC-MALS data provide a more robust analysis as there are a number of assumptions that need to be made in analysing the SDS–PAGE gels. The SEC-MALS approach described in this article would also be appropriate to study the effect of ionic strength, temperature and various cofactors (*S*-adenosyl methionine, *S*-adenosyl homocysteine, sinefungin, etc.) on the subunit stoichiometry and/or the ATP hydrolysis-triggered conformational switch to the sliding mode.

The subunit stoichiometry has important implications for the long-range intersite communication process and DNA cleavage. Our results clearly demonstrate that Type III REs work as monomeric non-classical helicase motors. As we did not observe any change in the subunit composition following ATP hydrolysis, the sliding competent form is the same species as the Res_1_Mod_2_ heterotrimer capable of recognition site binding. Furthermore, according to the ATP hydrolysis switch-driven DNA sliding model (28), upon formation of the collision complex, two endonuclease domains form the active DNA cleavage competent assembly, where a single endonuclease is necessary for the cleavage of each DNA strand, simplifying possible DNA cleavage schemes (12).

Under the reaction conditions used, Type III REs bind only a single copy of a recognition sequence per holoenzyme, despite the presence of two Mod subunits and therefore two Target Recognition Domains. The suggested DNA looping states (26,27) must involve two separate holoenzymes coming together, one at each recognition site, rather than a single enzyme binding two DNA simultaneously. Although we did not use a two-site DNA substrate in our SEC-MALS and native MS studies, we note that we did not obtain any evidence for the formation of any higher order species analogous to that in a DNA looping complex (e.g. a dimer of Res_1_Mod_2_ heterotrimers, each bound to one DNA).

Another implication of our observations is that it provides information for how dimeric methyltransferases may operate (23,38–41). As mentioned above, Type III REs bind only one copy of their recognition sites despite having two Target Recognition Domains per heterotrimeric enzyme, strongly suggesting an asymmetry within the two Mod subunits. We did not test the DNA binding stoichiometry of the isolated Mod_2_ dimer, however, we cannot rule out the possibility that the Res subunit binding induces the asymmetry. The observed DNA binding stoichiometry is also in agreement with the enzyme only methylating one strand of the recognition site (29,42,43). Other homodimeric methyltransferases can, however, form symmetrical dimers and methylate both strands of the DNA in one binding event (as opposed to the two events required of a monomeric system) (41). The exact nature of the geometry of the Type III holoenzyme awaits an atomic resolution crystal structure in complex with DNA.

One of the key aspects of the ATP-driven switch is that ATP hydrolysis drives a conformation change into a sliding competent state. Binding studies suggest that if this structure dissociated from DNA during sliding by exiting at a DNA end, the sliding conformation is retained for tens of seconds and is unable to rebind DNA. We interpret the decreased stability of the heterotrimer enzyme in the presence of sDNA upon the addition of ATP under the native MS conditions as an indication for this conformational difference.

Finally, in both the SEC-MALS and native MS we observed DNA cleavage despite the fact that the rubric states that Type III enzymes need two DNA sites in inverted repeat on the same DNA for nuclease activity. The cleavage seen here does not require direct connection between the sites. However, the sliding state must be initiated by ATP hydrolysis. We suggest that the sliding on DNA acts to not only report on the relative orientation of the sites, but elevates the local concentration of nuclease domains by a reduction in dimensionality. This necessity can be removed by elevating the concentration of ‘activated’ enzymes. Under normal conditions, the concentration of enzymes that dissociate from DNA in an activated state is too small to produce the protein–protein interactions with DNA-bound enzymes. With the short single-site DNA duplex used in this study (Figure 2A), there is a free DNA end close to the cleavage site. We have previously observed that EcoPI in an activated form can interact with DNA ends (44). It is possible that the elevated cleavage seen here may reflect the location of the recognition/cleavage sites. Further studies could be informative regarding how activated enzymes interact with enzymes bound at recognition sites within different substrates.

## SUPPLEMENTARY DATA

Supplementary Data are available at NAR Online.

## FUNDING

The Wellcome Trust [084086 to M.D.S. and 078794 to S.H.]; the Research Foundation Flanders [FWO G093811N to F.S.]; the Synapt G2 mass spectrometer by the Hercules Foundation – Flanders [AUHA/014/09 to F.S.]; financial support in the form of a PhD fellowship from UA (to A.B.); F.S. is a Francqui Research Professor at UA. Funding for open access charge: Wellcome Trust [084086 to M. D. S.].

*Conflict of interest statement*. None declared.

## Supplementary Material

Supplementary Data
