# Supplementary material for: Type III restriction endonucleases are heterotrimeric: comprising one helicase–nuclease subunit and a dimeric methyltransferase that binds only one specific DNA
Source: Nucleic Acids Res. 2014 Feb 6;42(8):5139–50. doi: 10.1093/nar/gku122 (PMC4005696; doi:10.1093/nar/gku122)
Supplement: Supplementary Data [file supp_42_8_5139__index.html]

Type III restriction endonucleases are heterotrimeric: comprising one helicase–nuclease subunit and a dimeric methyltransferase that binds only one specific DNA — Type III restriction endonucleases are heterotrimeric: comprising one helicase–nuclease subunit and a dimeric methyltransferase that binds only one specific DNA — Supplementary Data 

# Type III restriction endonucleases are heterotrimeric: comprising one helicase–nuclease subunit and a dimeric methyltransferase that binds only one specific DNA

## Supplementary Data

files

**Files in this Data Supplement:**

- Supplementary Data - pdf file
