# Supplementary material for: Type III restriction endonucleases are heterotrimeric: comprising one helicase–nuclease subunit and a dimeric methyltransferase that binds only one specific DNA
Source: Nucleic Acids Res. 2014 Feb 6;42(8):5139–50. doi: 10.1093/nar/gku122 (PMC4005696; doi:10.1093/nar/gku122)
Supplement: Supplementary Data [file supp_gku122_nar-03613-f-2013-File006.pdf]

SUPPLEMETARY DATA

Type III restriction endonucleases are heterotrimeric: comprising one helicase-nuclease subunit and a dimeric methyltransferase that binds only one specific DNA

Annika Butterer, Christian Pernstich, Rachel M. Smith, Frank Sobott, Mark D. Szczelkun and Júlia Tóth

SUPPLEMENTARY METHODS

SDS-polyacrylamide gel electrophoresis of Type III REs

3 µg of wt EcoP15I, wt EcoP15I from New England Biolabs, the biotinylated version of EcoP15I, the R534A helicase mutant of EcoP15I, wt EcoPI and wt PstII reduced with β-mercaptoethanol were run on an 8 % (w/v) SDS-polyacrylamide gel according to standard methods (45). The gel was stained with InstantBlue, a coomassie-based dye (Expedeon).

Analytical Ultracentrifugation

Wt EcoP15I was dialysed into standard assay buffer minus BSA (Materials and Methods). Samples were set up for sedimentation equilibrium runs (Beckman XL-A analytical ultracentrifuge) at 3 different concentrations, 0.38 µM (A_{280} = 0.1), 0.94 µM (A_{280} = 0.25) and 1.87 µM (A_{280} = 0.5) at room temperature (20.0 ± 1 °C). During centrifugation the absorbance of the samples was recorded at 230, 280 or 292 nm, depending on the concentration of the protein. Rotor speeds (6k, 7.5k and 10k rpm) were chosen according to the expected molecular weight for EcoP15I. Runs were continued until equilibrium was reached (≥ 16 h) and at least two scans were performed at each rotor speed. The precise concentration of the protein was determined from an initial absorbance reading on reaching 3k rpm. To determine the baseline absorbance, a final scan was recorded after 6 h of overspeeding at 40k rpm and readings were taken near the meniscus. Radial absorption scans were analyzed by global fitting using

Microcal Origin software (MicroCal) to Equation 1 describing the distribution of a single ideal species after sedimentation to equilibrium:

$$A_r = A_{r0} \times e^{\left[\frac{\omega^2}{2RT} M\left(1-\bar{v}\rho\right)\left(r^2-r_0{}^2\right)\right]} + B \qquad \text{(Equation 1)}$$

A_r and A_{r0} are the absorbance at radius r and at the reference r_0, ω the centrifugal speed, $\bar{v}$ the partial specific volume, ρ the solvent density and B is the baseline absorbance value. Depending on whether it is applied to a single or multiple species, M is either the molecular weight of that species or the mean weight-average molecular weight of all the species in the sample assuming that the mixture of species has varied mass but uniform $\bar{v}$ (46,47). However, the native MS and MALS data in the main manuscript are consistent with homogenous populations consisting of a single species.

TBE-polyacrylamide gel electrophoresis assays of DNA cleavage

Samples of Type III REs were pre-incubated with specific DNA duplexes in the absence or presence of ATP and, following 30 minute incubation at room temperature, separated using a Superdex 200 gel filtration column. Fractions containing DNA were subjected to native TBE-PAGE on 20 % (w/v) polyacrylamide gels at 4 °C to separate the intact and cleaved DNA species. The gels were stained with ethidium bromide to visualize the bands of the GeneRuler Ultra Low Range DNA ladder (Fermentas).

To determine the cleavage kinetics, reactions were set up under the SEC-MALS experimental conditions (5 μM sDNA, 2.5 μM wt EcoP15I and 1 mM ATP) in standard assay buffer. The reactions were initiated by adding the enzyme to the reaction mix. At timed intervals, aliquots were quenched by adding 1/5 volume of 5x loading dye (40 % (w/v) sucrose, 0.25 % (w/v) bromophenol blue, 100 mM EDTA pH 8.0) and heat-inactivated by heating to 80 °C for 5 minutes. The gels were then scanned for hexachlorofluorescein (Hex) fluorescence, and the band intensities analyzed by densitometry using the TL100 software (Non-linear Dynamics). Single (Equation 2, red line in Figure S4C) and double (Equation 3, blue line in Figure S4C) exponential functions with an offset were fit to the resulting data using GraphPad Prism (GraphPad Software).

$$y = \mathrm{A}_0.e^{-kt} + \mathrm{offset} \qquad \text{(Equation 2)}$$

$$y = A_{0(1)}.e^{-k_1 t} + A_{0(2)}.e^{-k_2 t} + \text{offset}$$ (Equation 3)

where A_0, $A_{0(1)}$ and $A_{0(2)}$ are amplitudes, k, k_1 and k_2 are the rate constant of the decay and t is time.

Stopped flow fluorescent assays of DNA dissociation

The kinetics of dissociation from DNA (Figure 4SC, grey line, right Y-axis) was measured as described previously (main text 28). In brief, 50 nM Hex-labelled 50-mer specific duplex (Figure 2A) was pre-incubated with 106 nM wt EcoP15I and rapidly mixed against 2 mM ATP and 10 μM unlabelled specific duplex in an SF61-DX2 stopped flow apparatus (TgK Scientific, UK) equipped with polarizing optics. The final concentrations were 25 nM sDNA, 53 nM wt EcoP15I and 1 mM ATP. The dissociation of the enzyme from the DNA was monitored by recording the fluorescence intensity change of the Hex label (λ_{ex} = 540 nm) through 550 nm cut-off filters (Schott OG550) in T-format. The fluorescence changes in the parallel and perpendicular plane were transformed into anisotropy change, which then was converted to the percentage of dissociation from the DNA by utilizing a binding titration curve (data not shown) (main text 28).

SUPPLEMENTARY FIGURES

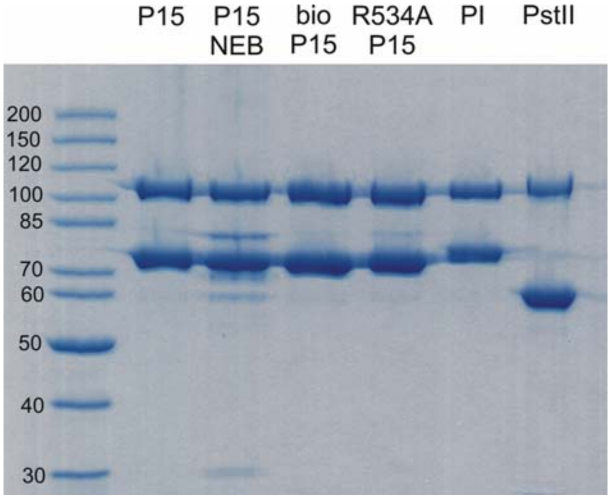

Figure S1 SDS-PAGE gel of the Type III restriction endonucleases used in the assays

The gel shows that the enzyme preparations are highly homogeneous and there is no significant level of proteolytic degradation detectable. The denatured enzymes run on the gel as two separate polypeptides corresponding to the expected subunit molecular weights of ~108-112 kDa (for Res) and ~64-74 kDa (for Mod). The molecular weight (kDa) of the bands of PageRuler Unstained protein ladder in lane one (Fermentas) are shown on the left hand side of the gel.

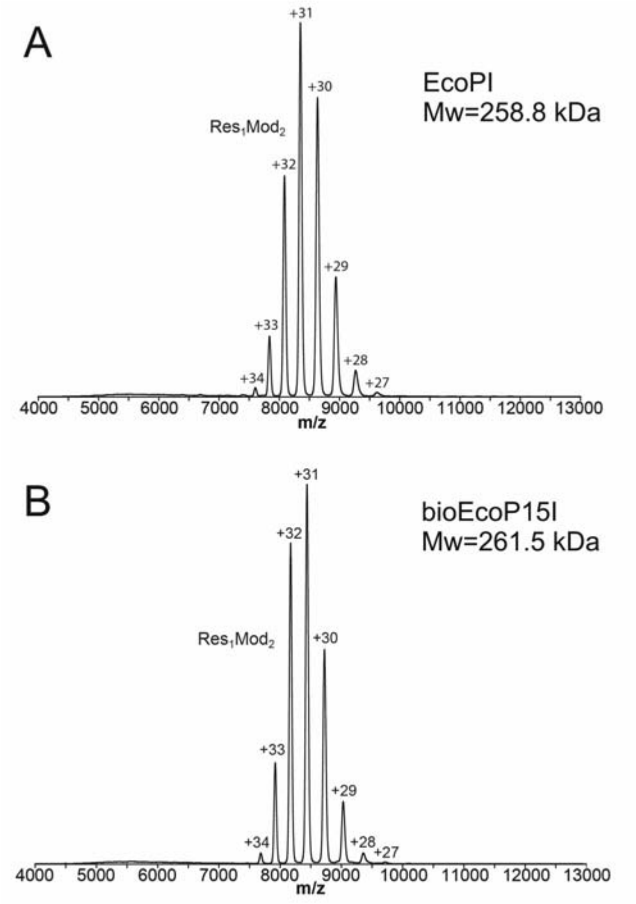

Figure S2 Native mass spectra of Type III restriction enzymes

Native mass spectra of wt EcoPI (**A**) and the biotinylated EcoP15I used in previous single molecule studies (main text 28) (**B**). The y-axis is relative intensity, scaled to the most intense peak in the spectrum, which is the 31+ charge state of the enzyme complex. The measured molecular masses (258.5 and 261.5 kDa, respectively) are in closest agreement with a Res_1Mod_2 subunit organization (see Table 1). This data also confirms that engineering an Avi -tag to the C-terminus of the Res subunit and labelling it with a biotin molecule does not interrupt or change the native stoichiometry.

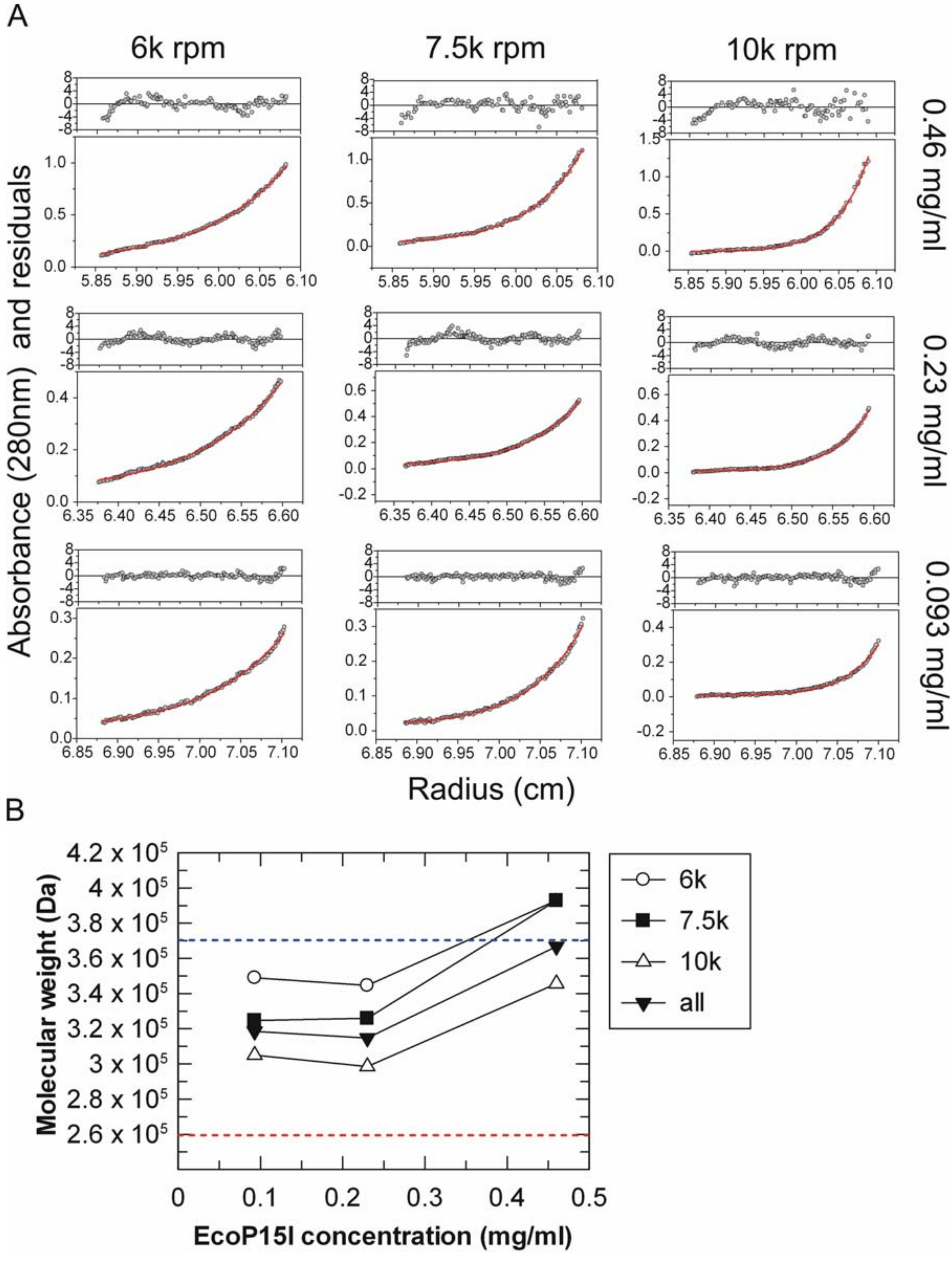

A
6k rpm
7.5k rpm
10k rpm
0.46 mg/ml
0.23 mg/ml
0.093 mg/ml
Absorbance (280nm) and residuals
Radius (cm)
B
Molecular weight (Da)
EcoP15I concentration (mg/ml)
6k
7.5k
10k
all

Figure S3 Analytical ultracentrifugation data for wt EcoP15I

(A) Analytical ultracentrifugation sedimentation equilibrium traces of wt EcoP15I at three different concentrations and at three different running speeds, as indicated. The red lines are the best fits to Equation 1, with the residuals shown above.

(B) Dependence of the calculated molecular weights on the protein concentration and the applied centrifugal speed. Increasing the protein concentration in the sample chambers increased the apparent molecular weight indicative of higher molecular weight aggregates forming in solution, whereas applying higher centrifugal forces to the samples resulted in lower apparent molecular weights. The dotted red and blue lines mark the theoretical molecular weights of the Res_1Mod_2 heterotrimer and the Res_2Mod_2 heterotetramer, respectively (Table 1).

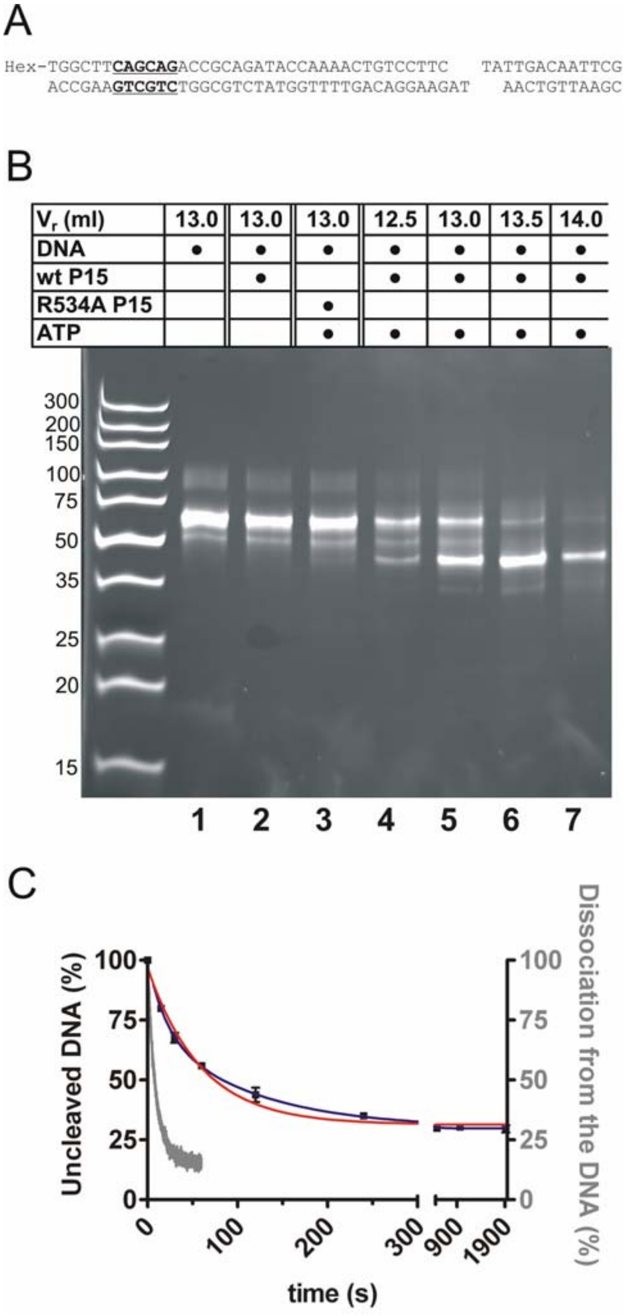

Figure S4 The specific 50-mer DNA duplex with a single recognition site is cleaved by EcoP15I in the presence of ATP under chromatography conditions

(A) Cleavage products of the 50-mer EcoP15I-specific DNA duplex (Figure 2A). Following site recognition and ATP hydrolysis, the DNA is cleaved by wt EcoP15I 25 and 27 bases downstream of the recognition site (5′-CAGCAG-3′, bold and underlined) resulting in a large and a small fragment of 37 and 11 base pairs, respectively, with a two-base overhang.

(B) TBE-polyacrylamide gel of gel filtration fractions of various samples of wt or R534A EcoP15I-sDNA cleavage reactions. The composition of the samples and midpoint retention volumes of the fractions (V_r) are shown in the table above the gel. Lanes 1, 2, 3 and 4-7 represent separate chromatography runs. Cleavage of the 50-mer specific DNA was observed only in samples with wt EcoP15I in the presence of ATP. The 11bp fragment was not observed on the ethidium stained gels probably due to detection sensitivity limits.
(C) Cleavage time-course of the specific 50-mer duplex by wt EcoP15I under the SEC-MALS reaction conditions compared to DNA dissociation at the same ATP concentration of 1 mM. The data was fitted to a single exponential (Equation 2, red) or to a double exponential (Equation 3, blue). The kinetics of dissociation from the specific DNA is plotted in grey (right Y-axis). The DNA cleavage time course is significantly slower than the dissociation time course, suggesting that enzymes need to have dissociated from the DNA in order to become activated to cleave the DNA under these conditions (see main text).

SUPPLEMENTARY REFERENCES

45. Laemmli, U.K. (1970) Cleavage of structural proteins during the assembly of the head of bacteriophage T4. *Nature*, **227**, 680-685.
46. Cantor, C.R. and Schimmel, P.R. (1980) Techniques for the Study of Biological Structure and Function. *Biophysical Chemistry, Part II,* W.H. Freeman & Company, San Francisco
47. Tanford, C. (1961) *Physical Chemistry of Macromolecules*. John Wiley and Sons, New York
